# Aurora-A/ERK1/2/mTOR axis promotes tumor progression in triple-negative breast cancer and dual-targeting Aurora-A/mTOR shows synthetic lethality

**DOI:** 10.1038/s41419-019-1855-z

**Published:** 2019-08-13

**Authors:** Wenfeng Zhang, Ding Xia, Zhangyun Li, Tao Zhou, Tingting Chen, Zhengping Wu, Weihua Zhou, Zilun Li, Longkun Li, Jie Xu

**Affiliations:** 1 Department of Infectious Disease, the First Affiliated Hospital, Nanchang University, Nanchang, PR China; 2 Department of Urology, Tongji Hospital, Tongji Medical College, Huazhong University of Science and Technology, Wuhan, PR China; 3 Department of Oncology, the Third Hospital of Nangchang, Nanchang, PR China; 4 Department of Urology, the Second Affiliated Hospital, Third Military Medical University (Army Medical University), Chongqing, PR China; 5 Division of Cell and Radiation Biology, Department of Radiation Oncology, University of Michigan, Ann Arbor, MI USA; 6 Division of Vascular Surgery, the First Affiliated Hospital of Sun Yat-Sen University, Guangzhou, PR China

**Keywords:** Targeted therapies, Cell invasion

## Abstract

Triple-negative breast cancer (TNBC), defined as a tumor subtype that lacks ER, PR, and HER2, shows a poor prognosis due to its aggressive tumor biology and limited treatment options. Deregulation of Aurora kinase A (Aur-A), a member of the mitotic serine/threonine Aurora kinase family, and overactivation of the mTOR pathway commonly occur in multiple cancer types. We previously found that Aur-A activated the mTOR pathway and inhibited autophagy activity in breast cancer cell models. Whether and how Aur-A regulates mTOR in TNBC are still unclear. Here, we found that Aur-A and p-mTOR are highly expressed and positively associated with each other in TNBC cells and tissues. Inhibition or knockdown of Aur-A decreased p-mTOR and suppressed cell proliferation and migration, whereas overexpression of Aur-A increased p-mTOR levels and promoted cell proliferation and migration, which was significantly abrogated by simultaneous silencing of mTOR. Intriguingly, overexpression of Aur-A enhanced the expression of p-mTOR and p-ERK1/2, and silencing or inhibition of ERK1/2 blocked Aur-A-induced p-mTOR. However, silencing or inhibition of mTOR failed to reverse Aur-A-induced ERK1/2, indicating that Aur-A/ERK1/2/mTOR forms an oncogenic cascade in TNBC. We finally found that double inhibition of Aur-A and mTOR showed significant synergistic effects in TNBC cell lines and a xenograft model, indicating that Aur-A and mTOR are potential therapeutic targets in the TNBC subtype.

## Introduction

Breast cancer is one of the leading causes of cancer-related death among adult women worldwide^1^. The management and prognosis of breast cancer is largely dependent on the expression of estrogen receptor (ER), progesterone receptor (PR), and human epidermal growth factor receptor 2 (HER2)^2–4^. Triple-negative breast cancer (TNBC), characterized by negative expression of ER, PR, and HER2, comprises ~15% of all breast cancers^5^, and shows a poor prognosis due to the high rates of local and systemic relapse and the lack of available endocrine or molecularly targeted treatments^6–8^.

In recent years, molecular-targeted therapies have attracted increasing attention in TNBC treatment. As a first-line treatment for metastatic breast cancer, the combination of antiangiogenic inhibitor bevacizumab with taxol prolongs survival of TNBC patients^9^. EGFR protein levels are higher in TNBC than the other subtypes of breast cancers, and the combination of a monoclonal antibody targeting EGFR with cisplatin extends both disease-free and overall survival^10^. Furthermore, PARP (poly real (adenosine diphosphate-ribose) polymerase) enzyme, which is mainly involved in the repair of the base excision after DNA damage^11^, has been demonstrated as a novel clinical target for TNBC, and the combination of PARP inhibitor iniparib with other chemotherapeutic drugs significantly prolongs the survival of TNBC patients^12^. As a result, increasing studies focus on exploring new therapeutic targets and developing their corresponding small molecule inhibitors as a single or combination therapy in TNBC treatment.

Aur-A (also known as STK15, BTAK, and Aurora-2), a member of the mitotic serine/threonine Aurora kinase family, is essential in accurate timing of mitosis and maintenance of bipolar spindles^13,14^. Aur-A has been subsequently reported as an oncogenic factor: (1) it was amplified and/or overexpressed in multiple cancers, including breast, ovarian, and hepatic carcinomas^15^; (2) forced Aur-A expression resulted in NIH3T3 fibroblast oncogenic transformation and induced aneuploidy in breast cancer cells^16^; and (3) Aur-A interacts with and modifies the functions of several key cancer-associated molecules, such as the inactivation of BRACA^17,18^ and p53^19,20^, leading to tumorigenesis and tumor progression. Inhibition of Aur-A kinase by its pan- or specific-inhibitors suppressed cell proliferation, growth, and progression in multiple cancer types^21–24^. Furthermore, selective Aur-A kinase inhibitor Alisertib (MLN8237) has been used in clinical trials^25–27^. However, the single-agent treatment of Alisertib is still limited by the modest side effects, such as febrile neutropenia, anemia, thrombocytopenia, and neutropenia, etc^28^. Combination of Alisertib with other chemotherapeutic agents or small molecular inhibitors may decrease the toxicity caused by single-agent treatment and enhance its anticancer effects^27^.

Our recent studies found that Aur-A activates mammalian target of rapamycin (mTOR) pathway in breast cancer cell models and inhibits autophagy activity, which is not dependent on the classical PI3K-AKT1 pathway^29^. Thus, the mechanism by which Aur-A regulates mTOR and whether dual inhibition of Aur-A and mTOR enhances the effects of single inhibitors in TNBC remain unknown. In this study, we found that Aur-A and p-mTOR are overexpressed and positively associated with each other in TNBC cell lines and human tissues. Aur-A, via activation of the ERK1/2 pathway, positively regulates p-mTOR to promote cell proliferation and growth in TNBC. Dual inhibition of Aur-A and mTOR shows significant synergistic effects in vitro and in vivo in TNBC models.

## Results

### Expression of Aur-A and mTOR in breast cancer cell lines and TNBC tissues

**Fig. 1:**
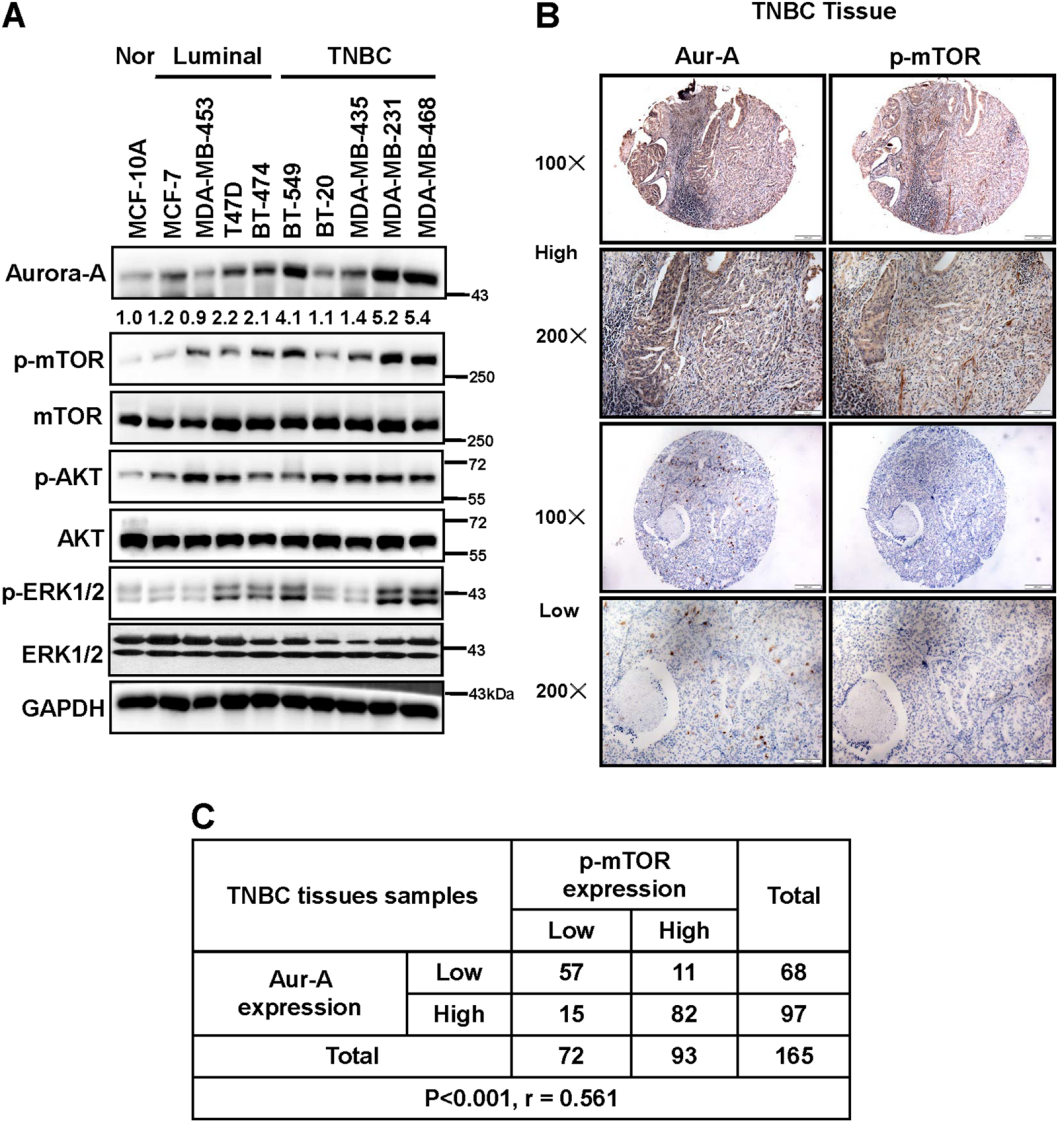
Expression of Aur-A and mTOR in breast cancer cell lines and TNBC human tissues. **a** Basal levels of Aur-A, p-mTOR, mTOR, and other proteins in breast cancer cell lines. **b** Aur-A and p-mTOR expression in TNBC human tissues. TNBC tissue microarrays were stained with Aur-A and p-mTOR and then photographed (Scale bars, 100 μm). **c** Association analysis of Aur-A and p-mTOR in TNBC. Data were analyzed by using SPSS software (*P* < 0.001, Pearson’s test)

Our previous study showed that Aur-A suppressed autophagy by activating the mTOR pathway^29^. To confirm the association of Aur-A and mTOR in the TNBC subtype, we first detected the expression of the two proteins and other related proteins in five TNBC cell lines (BT-549, BT-20, MDA-MB-435, MDA-MB-231, and MDA-MB-468), along with four non-TNBC breast cancer cell lines (MCF-7, MDA-MB-453, T47D, and BT-474) and a noncancerous breast epithelial cell line (MCF-10A) as controls. We previously showed that Aur-A expression was higher in TNBC cells and tissues compared with the non-TNBC subtype^30^. Compared with the normal breast epithelial cell line or non-TNBC cell lines, TNBC cell lines consistently present with much higher Aur-A expression, which is strongly elevated in three (BT-549, MBA-MD-468, and MBA-MD-231) of the five TNBC cell lines (Fig. 1a). We also found that p-mTOR and p-ERK1/2, but not p-Akt, show a significant positive correlation with Aur-A in the TNBC subtype (Fig. 1a). We also confirm the association between Aur-A and p-mTOR in TNBC tissues by using IHC staining. Among 165 TNBC human tissues, both Aur-A (97/165: 58.8%) and p-mTOR (93/165: 56.4%) were elevated and showed a positive correlation (Fig. 1b, c; *P* < 0.001, *r* = 0.561, Pearson’s x^2^-test). Thus, we confirmed that Aur-A shows a positive association with p-mTOR in the TNBC subtype.

### Aur-A promotes cell proliferation and invasion through activation of mTOR in TNBC cell lines

**Fig. 2:**
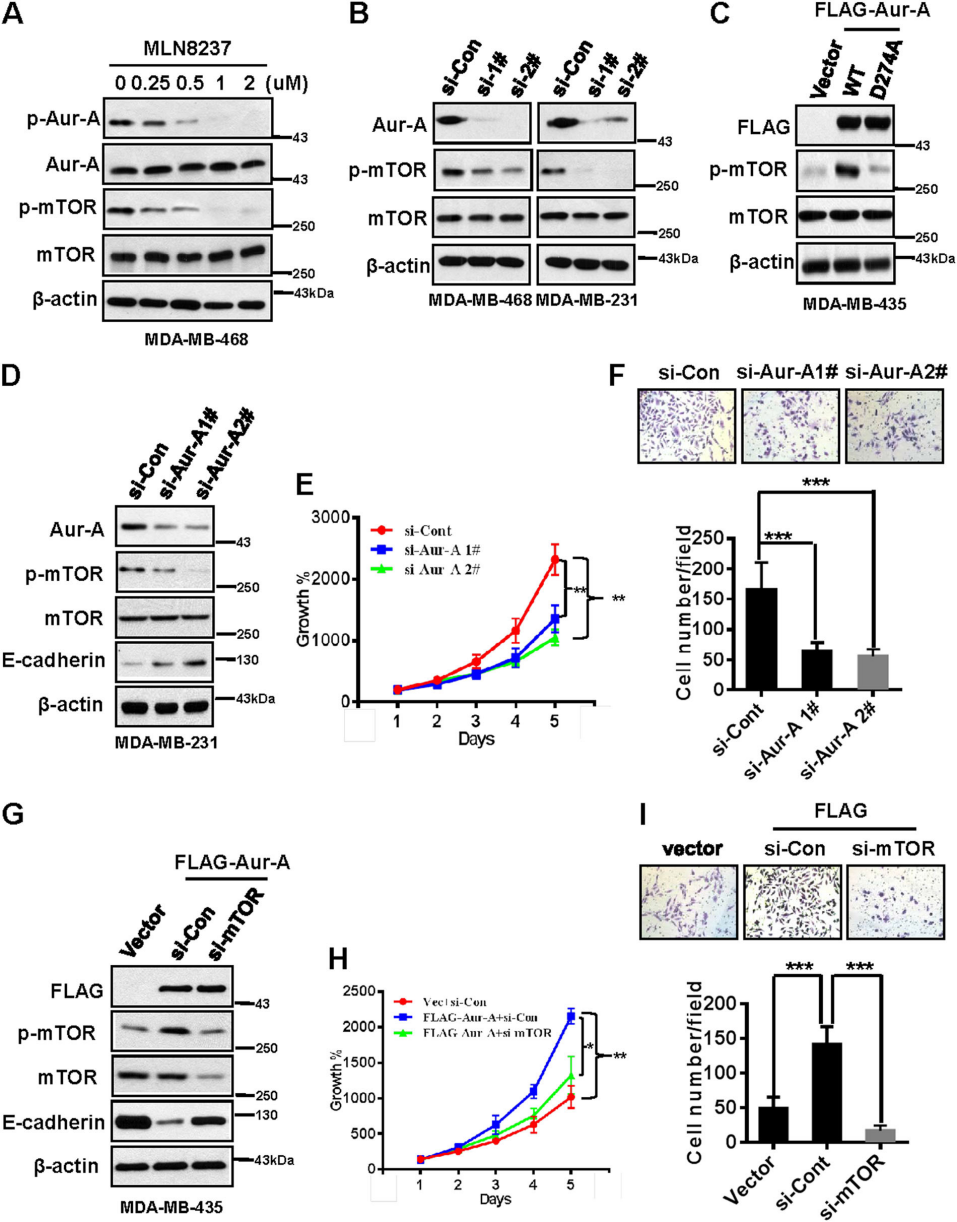
Aur-A promotes cell proliferation and invasion through activation of mTOR in TNBC cell lines. **a**, **b** Inhibition or silencing of Aur-A decreases both p-Aur-A and p-mTOR in TNBC cells. Cells were incubated with indicated doses of MLN8237 (0, 0.25, 0.5, 1, and 2 μM) for 48 h or transfected with siRNAs targeting Aur-A, followed by IB analysis. **c** Overexpression of Aur-A-WT increases p-mTOR, but its dead mutant decreases p-mTOR. Cells were transfected with indicated plasmids and collected for IB assay after transfection. **d**–**f** Silencing Aur-A inhibits cell proliferation and suppresses migration by decreasing p-mTOR. Cells were transfected with siRNAs targeting Aur-A and then collected for IB assay (**d**), ATPlite assay (**e**), or transwell migration assay (**f**) (mean ± SD; **P < 0.01). **g**–**i** Overexpression of Aur-A promotes cell proliferation and migration by activating mTOR and simultaneously silencing mTOR abrogates these effects. Cells were initially transfected with vector control or FLAG-tagged Aur-A first and then silenced using siRNAs targeting control or mTOR, followed by IB (**g**), ATPlite (**h**), and transwell migration (**i**) assays (mean ± SD; *P < 0.05; **P < 0.01)

We then tried to determine the biological functions of Aur-A and mTOR in two TNBC models, with one showing the highest expression of Aur-A (MDA-MB-468 or -231) and another showing moderate expression of Aur-A (MDA-MB-435 or BT-20). We treated MDA-MB-468 (Fig. 2a) or MDA-MB-231 (Fig. S1A) cells with MLN8237, a specific inhibitor of Aur-A kinase^26,31^, and confirmed that MLN8237 inhibited Aur-A by reducing its autophosphorylation, leading to the decrease of p-mTOR in a dose-dependent manner. Silencing Aur-A caused a significant decrease of p-mTOR in the two Aur-A-highly-expressed TNBC cell lines (Fig. 2b), further indicating that Aur-A positively regulates p-mTOR levels in TNBC cell lines. We then exogenously overexpressed Aur-A-WT or its kinase dead mutant -D274A in MDA-MB-435 (Fig. 2c) or BT-20 (Fig. S1B) cell lines, which show moderate Aur-A expression compared with MDA-MB-468 and MDA-MB-231. We found that p-mTOR was accumulated in cells transfected with Aur-A-WT, whereas attenuated in cells transfected with kinase dead Aur-A-D274A.

We previously showed that Aur-A overexpression promoted cell invasion or migration in TNBC^30^. We then sought to determine whether Aur-A mediates it via the mTOR pathway. MDA-MB-231 (Fig. 2d) or MDA-MB-468 (Fig. S1C) cells were first transfected with si-Cont or si-Aur-A using two different oligos, and western blot results showed that both of the oligos caused a significant decrease of Aur-A (50–70%), followed by a decrease of p-mTOR and increase of E-cadherin migration marker. ATPlite (Fig. 2e or S1D) and transwell assays (Fig. 2f or S1E) further demonstrated that silencing of Aur-A significantly inhibited cell proliferation and invasion in TNBC cells. To further confirm this, MDA-MB-435 (Fig. 2g–i) or BT-20 cells (Fig. S1F–H) were transfected with vector control or FLAG-tagged Aur-A, followed by combination with si-cont or si-mTOR. Our data showed that overexpression of Aur-A caused the increase of p-mTOR and decrease of E-cadherin protein levels, resulting in enhanced cell proliferation and migration. Simultaneous knockdown of mTOR significantly abrogated the effects caused by Aur-A overexpression. Our data demonstrate that Aur-A promotes TNBC cell proliferation and migration via activation of the mTOR pathway.

### Aur-A positively regulates the activity of mTOR through the ERK1/2 pathway

**Fig. 3:**
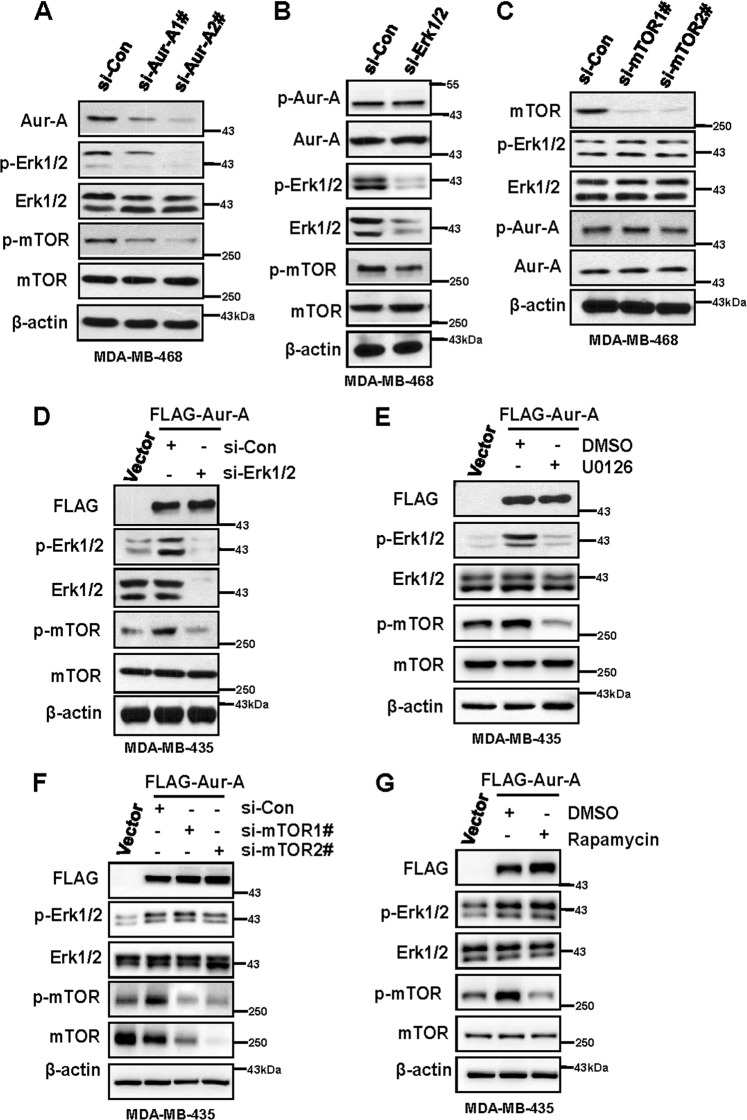
Aur-A activates mTOR through ERK1/2 pathway. **a** Silencing Aur-A decreased p-mTOR and p-ERK1/2. Cells were transfected with siRNAs targeting control or Aur-A, followed by the IB assay 48 h post transfection. **b**, **c** Silencing ERK1/2 decreased p-mTOR, but silencing mTOR failed to change the level of p-ERK1/2. Cells were transfected with siRNAs targeting control or ERK1/2 (**b**) or mTOR (**c**), followed by the IB assay. **d**, **e** Silencing or inhibition of ERK1/2 significantly rescued the increase of p-mTOR caused by Aur-A overexpression. Cells were transfected with vector or FLAG-Aur-A, along with transfection of siRNAs targeting control or ERK1/2 (**d**), or treatment with DMSO or U0126 (**e**). **f**, **g** Silencing or inhibition of mTOR failed to rescue the increase of p-ERK1/2 caused by Aur-A overexpression. Cells were transfected with vector or FLAG-Aur-A, along with transfection of siRNAs targeting control or mTOR (**f**), or treatment with DMSO or rapamycin (**g**)

We previously found that Aur-A, via inducing mitogen-activated protein kinase (MAPK) phosphorylation, promoted epithelial-mesenchymal transition and invasion in nasopharyngeal carcinoma (NPC)^32^. In addition, components of the Ras-ERK pathway (Ras, Raf, ERK, and RSK) also positively regulate the PI3K-mTOR pathway^33^. Thus, we further determined whether Aur-A/ERK1/2/mTOR forms a cascade axis in TNBC. MDA-MB-468 cells were transfected with siRNAs targeting control or Aur-A, and the data demonstrated that silencing Aur-A decreased p-mTOR and p-ERK1/2 (Fig. 3a). We further found that silencing ERK1/2 alone in MDA-MB-468 cells downregulated p-mTOR, but not p-Aur-A (Fig. 3b). Furthermore, silencing mTOR failed to change the levels of both p-ERK1/2 and p-Aur-A (Fig. 3c). We had similar findings when cells were treated with mTOR inhibitor rapamycin or ERK1/2 inhibitor U0126 (Fig. S2), indicating that the Aur-A/ERK1/2/mTOR axis indeed forms an oncogenic cascade in the TNBC subtype.

We further transfected MDA-MB-435 cells with vector control or FLAG-Aur-A, along with simultaneous silencing of ERK1/2 or control. The result showed that overexpression of Aur-A caused the accumulation of p-ERK1/2 and p-mTOR, which was abrogated in a large extent by simultaneous silencing of ERK1/2 (Fig. 3d) or treatment with ERK inhibitor U0126 (Fig. 3e). Intriguingly, silencing or inhibition of mTOR in FLAG-Aur-A-overexpressed cells could not block Aur-A-induced p-ERK1/2 (Fig. 3f, g), further suggesting that Aur-A regulates mTOR activity through the ERK pathway.

### Dual inhibition of Aur-A and mTOR shows synergistic lethality in vitro

**Fig. 4:**
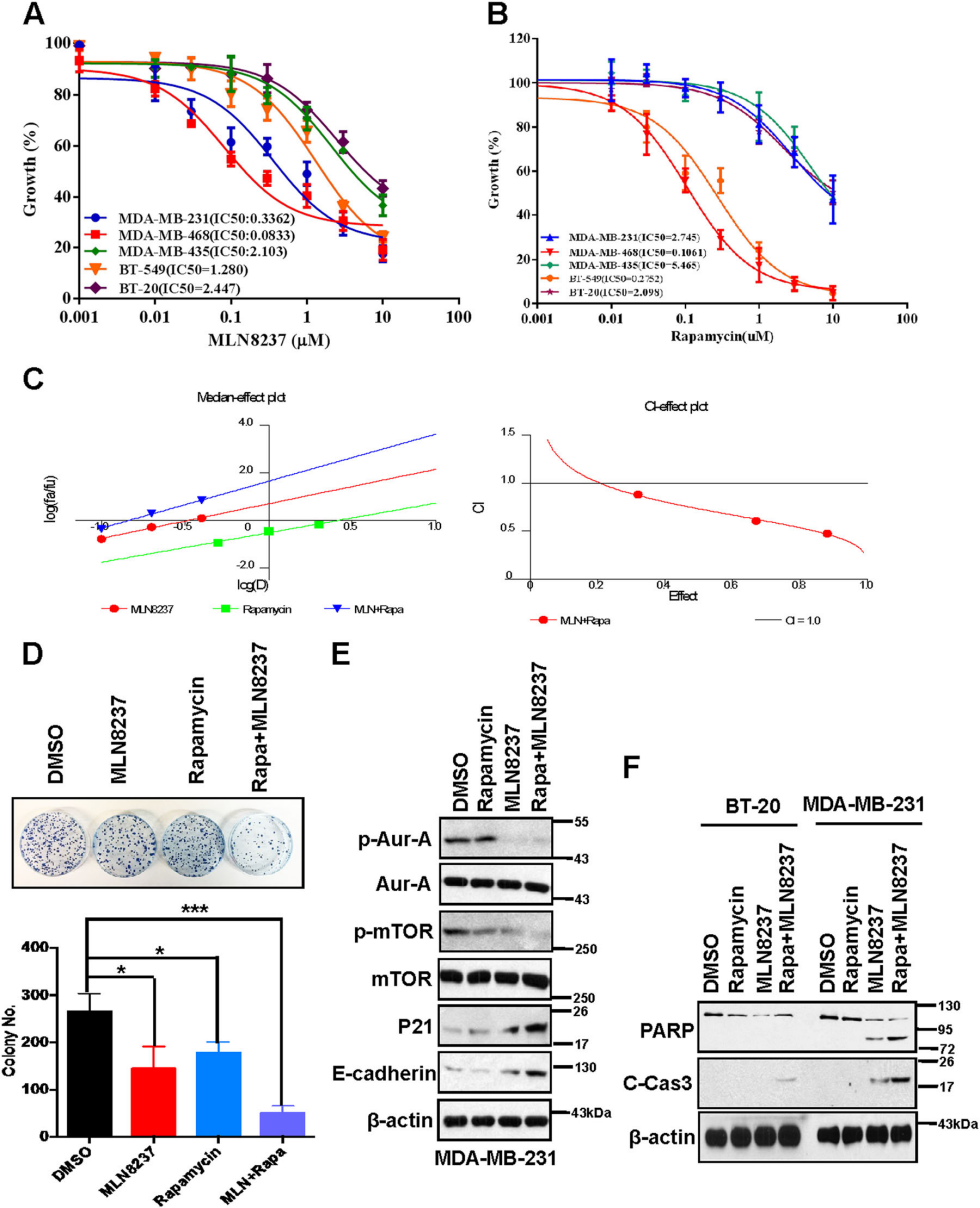
MLN8237 and rapamycin show synergistic effects in TNBC cell lines. **a**, **b** IC50 values of MLN8237 and rapamycin in five TNBC cell lines. Each cell line was treated with the indicated concentrations of MLN8237 (0.019, 0.039, 0.078, 0.156, 0.312, 0.625, 1.25, 2.5, 5, and 10 μM) (**a**) or rapamycin (0.019, 0.039, 0.078, 0.156, 0.312, 0.625, 1.25, 2.5, 5 and 10 μM) (**b**) for 48 h, followed by the ATPlite assay, IC50 of the two compounds were determined by GraphPad Prism5 software. **c** MLN8237 and rapamycin show synergistic effects in MDA-MB-231 cells. CI-effect plots and median effect plots were generated using CalcuSyn software. The points a, b, and c represent CI values for the combinations 0.1, 0.2, and 0.4 μM MLN8237 with 0.5, 1.0, and 2.0 μM rapamycin in a constant ratio, respectively. **d** Combination of MLN8237 and rapamycin inhibits cell colony formation in TNBC cells. MDA-MB-231 cells were treated with MLN8237 (0.2 μM) or rapamycin (1.0 μM) alone or combination of the two compounds, followed by the colonogenic assay. The data were expressed as mean ± S.D. (*P < 0.05, **P < 0.01; Student’s *t*-test) compared with control groups. **e**, **f** Dual inhibition of Aur-A and rapamycin suppressed cell proliferation and induced apoptosis in TNBC cells. Cells were treated with MLN8237 (0.2 μM) or rapamycin (1.0 μM), or combination of the two compounds, followed by the IB assay with indicated antibodies

Overexpression of Aur-A is associated with tumor growth and poor prognosis^22,24^, and its pan- or specific-inhibitors have been used in clinic trials^25,26^. However, drug toxicity and resistance are still obstacles for its development in clinic^28^. Combination with inhibitors targeting other pathways may be helpful to reverse drug resistance or minimize the toxicities caused by Aur-A inhibitors. To determine the effects of dual inhibition of mTOR and Aur-A in TNBC cell models, we first identified the IC50 of MLN8237 and mTOR inhibitor rapamycin in different TNBC cell lines. Both MLN8237 (IC50 = 0.0833 μM) and rapamycin (IC50 = 0.1061 μM) monotherapy inhibited cell proliferation significantly in MDA-MB-468 (Fig. 4a, b) and showed less effects in MDA-MB-435 and MDA-MB-231 cells. We then chose the MDA-MB-231 cell line for the following combination experiments.

Both CalcuSyn software and Jin’s formula were used as previously described to determine the synergy of the two agents^31,34^. MDA-MB-231 cells were cultured with combinations of the two drugs at different doses but in a constant ratio (MLN8237 to rapamycin: 0.1–0.5 μM, 0.2–1 μM, and 0.4–2 μM, respectively) for 48 h. The combination of 0.1 μM MLN8237 with 0.5 μM rapamycin in MDA-MB-231 cells inhibited cell proliferation by 32.0%, compared with monotherapy of MLN8237 by 15.1% or rapamycin by 11.2%, indicating synergism (CI = 0.886; *Q* = 1.31; Fig. 4c). Escalating doses, i.e., cotreatment with 0.2 μM MLN8237 and 1 μM rapamycin (CI = 0.607; *Q* = 1.27) or 0.4 μM MLN8237 and 2.0 μM rapamycin (CI = 0.477; *Q* = 1.21), show synergetic effects in MDA-MB-231 cells (Fig. 4c). Furthermore, combination MLN8237 with rapamycin significantly inhibited the clonogenic survival in MDA-MB-231 TNBC cell line (MLN8237 or rapamycin vs. MLN8237 + rapamycin: *P* < 0.01; Fig. 4d), indicating that combination of the two agents significantly inhibits cell proliferation or growth, which was further demonstrated by the increase of p21 (Fig. 4e). In addition, the nature of suppression in cell proliferation or growth upon MLN8237 and rapamycin combination was via induction of apoptosis (Figs. 4f and S3), as evidenced by the dose-dependent increased cleavage of PARP and caspase-3.

### Dual inhibition of Aur-A and mTOR shows synergistic lethality in vivo

**Fig. 5:**
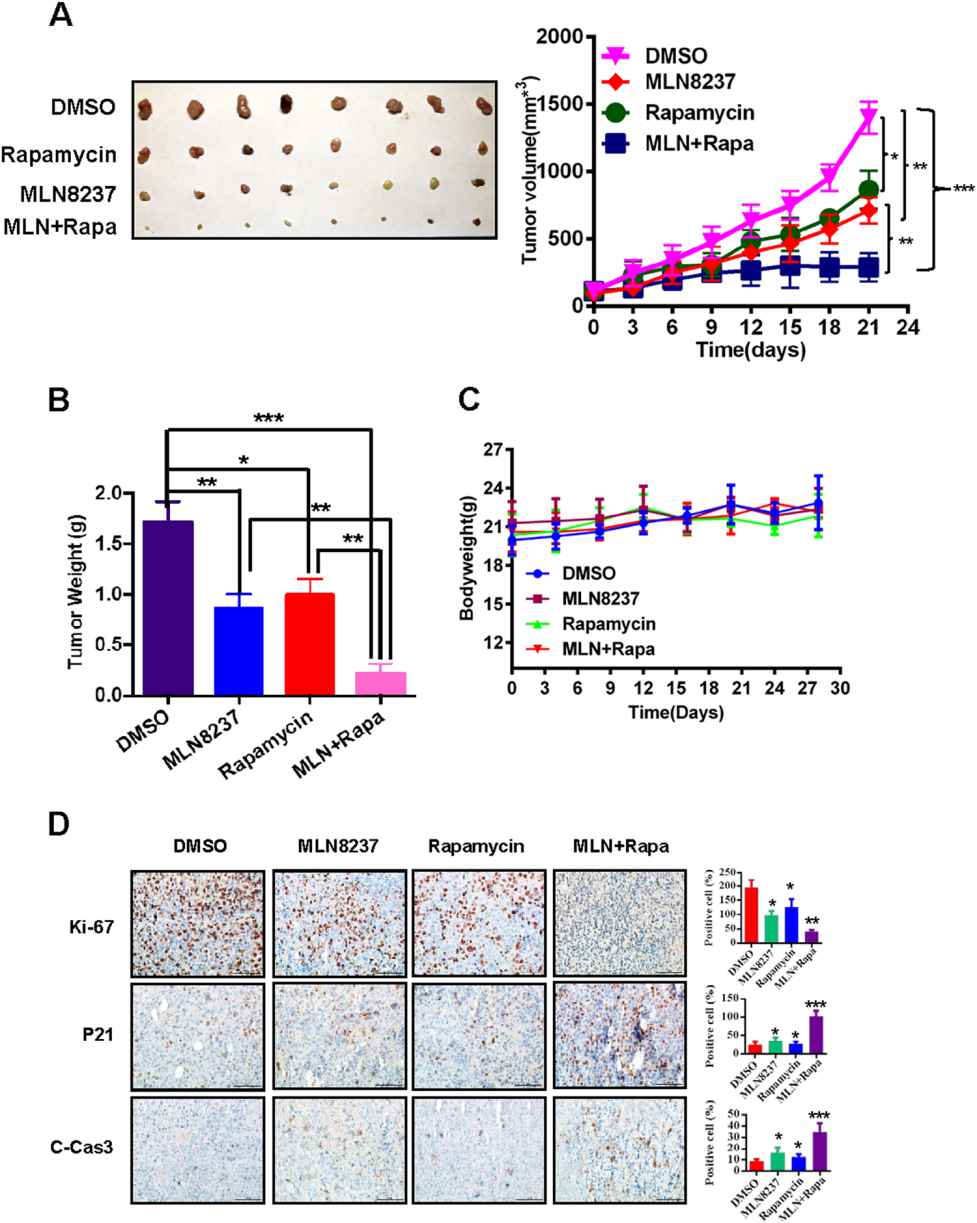
MLN8237 and rapamycin synergistically inhibit TNBC tumor growth in xenograft model. **a**, **b** Synergistic antitumor activity of MLN8237 and rapamycin in the MDA-MB-231 xenograft model. MDA-MB-231 cells were injected subcutaneously into both flank sides of nude mice. The mice were randomized when the tumor size reached 100 mm^3^ and were treated as follows: vehicle, *n* = 5; MLN8237 (30 mg/kg/days for 5 days/week for 4 weeks), *n* = 5; rapamycin (3.75 mg/kg/days for 5 days/week for 4 weeks), *n* = 5; MLN8237 + rapamycin, *n* = 5. The tumor growth was monitored and growth curve was plotted (**a**) and tumors were harvested and photographed (**b**). Data represent the mean ± SEM (*p < 0.05, **p < 0.01; Student’s *t*-test). **c** Both of the compounds were well tolerated in mice. Body weight was measured during the treatment and plotted (mean ± SEM). **d** Immunohistochemical staining of xenograft tumor tissues. Tumor tissues from four groups of mice were fixed, sectioned, and stained with indicated antibodies. Scale bars: 100 μm. Shown are mean ± SEM, *P < 0.05; **P < 0.01

We finally validate the above in vitro findings by using an in vivo xenograft model. A MDA-MB-231 xenograft model was established and treated as described in “Materials and methods” section. Consistent with the in vitro results, the combination of MLN8237 and rapamycin suppressed tumor growth significantly more than single-agent treatment (Fig. 5a, b; MLN8237 or rapamycin vs. MLN8237 + rapamycin: *P* < 0.01). The effect on normal tissues was minimal, if at all, as reflected by relatively unchanged body weight during drug treatment (Fig. 5c). Importantly, our IHC staining of tumor tissues revealed that compared with MLN8237 or rapamycin single-agent treatment, combination of the two agents more significantly inhibited cell growth (decrease of Ki-67 and increase of p21) and induced apoptosis (increase of cleavage caspase-3) (MLN8237 or rapamycin vs. MLN8237 + rapamycin: *P* < 0.01; Fig. 5d). Collectively, the results from both in vitro cell culture and in vivo xenograft models coherently demonstrate that the combination of MLN8237 and rapamycin more significantly inhibits cell growth and survival than single-agent treatment, with less effect on normal tissues, indicating that combination of the two agents serves as a promising strategy to conquer drug resistance caused by Aur-A inhibitor single-agent treatment.

## Discussion

Aur-A has been found to be overexpressed and diffusely localized in both the nucleus and cytoplasm^35^, indicating that it promotes tumorigenesis through distinct mechanisms. Indeed, Aur-A phosphorylates tumor suppressor RASSF1A at Thr202 and/or Ser203, inhibiting RASSF1A-mediated growth suppression in human cancers^36^. Overexpression of cyclin B2 significantly accelerates centrosome separation and leads to aneuploidy and tumorigenesis due to Aur-A-mediated hyperactivation of PLK1^37^. In addition, overexpression of Aur-A is associated with abrogation of DNA damage-induced apoptotic response and spindle assembly checkpoint override in cancer cells by phosphorylation of p73 at Ser235^38^. Aur-A has also been implicated in activation of NF-kB signaling by physical interactions with IKK kinases (IKKa and IKKβ) to trigger tumorigenesis and drive chemoresistance in AML^39,40^. We previously showed that Aur-A overexpression enhanced mammary cell migration by dephosphorylation and activation of cofilin, which facilitates actin reorganization and polymerization^41^. Furthermore, Aur-A promotes cell survival by suppressing autophagy in breast cancer models. In either nutrient deprivation or normal conditions, the overexpression of Aur-A inhibited autophagy by activating mTOR signaling, which was not dependent on the PI3K-AKT1 pathway^29^, but on the glycogen synthase kinase 3β pathway^42^.

In the present study, we further aimed to determine the association and biological functions of Aur-A/mTOR in a TNBC model. We found that both Aur-A and p-mTOR were highly expressed and positively associated with each other in TNBC cell lines and patient tissues (Fig. 1). Moreover, Aur-A promoted cell proliferation and invasion through activation of mTOR (Fig. 2). We found that Aur-A/mTOR did not significantly correlate with p-AKT, but more positively associated with the ERK1/2 pathway (Fig. 1a), suggesting a potential cross talk between Aur-A/mTOR and ERK1/2 in TNBC. We previously showed that Aur-A promoted epithelial-mesenchymal transition and invasion by MAPK phosphorylation in NPC cells^32^. Moreover, PI3K-mTOR could be positively regulated by the Ras-ERK pathway (Ras, Raf, ERK, and RSK)^33^. Herein, we found that Aur-A overexpression increased, whereas silencing decreased the expression of both p-mTOR and p-ERK1/2 (Fig. 3). While Aur-A overexpression increased the level of p-mTOR, simultaneous silencing or inhibition of ERK1/2 could partially abrogate it. However, silencing or inhibition of mTOR failed to block Aur-A-induced expression of p-ERK1/2, indicating that Aur-A/ERK1/2/mTOR forms an oncogenic cascade in TNBC model.

Numerous Aurora kinase inhibitors have been developed due to the important roles of Aur-A kinase in tumorigenesis. Alisertib (MLN8237), an investigational, oral, selective, small-molecule inhibitor of Aur-A kinase, shows preclinical activity against a broad range of tumor types^43^. Based on this encouraging preclinical activity, single-agent Alisertib has been used for multiple cancers in clinical trials^25,26^. In a recent study, the recommended phase II dose of alisertib, in combination with rituximab and/or vincristine, was well tolerated and showed activity in non-GCBDLBCL^27^. Although promising, selective Aur-A inhibitors are still limited by their mechanism-based toxicities. For example, Alisertib shows a modest anti-leukemic activity^28^. ENMD-2076, another small-molecule inhibitor of Aurora and angiogenic kinases, has meaningful clinical benefit in a small subset of patients with previously treated metastatic TNBC, but the 6-month CBR was only 16.7% (95% CI, 6–32.8%), and treatment by this inhibitor also resulted in mechanism-based toxicities, most commonly hypertension, fatigue, and diarrhea^44^. Recently, a preclinical study indicated that mTOR pathways were enriched in TNBC PDX models, which is associated with acquired resistance to ENMD-2076 treatment, in which case the combination of Aurora inhibitors with mTOR inhibitors would be a promising therapeutic strategy in TNBC^45^.

mTOR, consisting of mTORC1, which directly phosphorylates and activates S6 kinase and 4E-binding protein 1, and mTORC2, which phosphorylates AKT, plays an important role in cell growth, proliferation, survival, and metabolism^46^, serving as an attractive anticancer therapeutic target^47^. rapamycin, which blocks the mTORC1 complex, has been demonstrated to have variable antitumor effects in preclinical models and is currently under clinical investigation^48^. mTOR and Aur-A pathways are commonly deregulated in uterine leiomyosarcoma and their concomitant inhibition by rapamycin and MLN8237 abrogates the proliferation of uterine leiomyosarcoma cells^49^. Here, we also found that dual inhibition of Aur-A and mTOR, compared with monotherapy, inhibited more cell proliferation, migration, and survival in vitro and in vivo in TNBC models (Fig. 5).

In summary, our findings suggest that Aur-A deregulation and subsequent activation of the mTOR pathway plays an important role in TNBC tumorigenesis and provides supporting justification for clinical trials to evaluate combined Aur-A and mTOR inhibitors to enhance the anti-TNBC effects observed with single-agent inhibitor alone.

## Materials and methods

### Reagents and cell cultures

MLN8237, U0126, and rapamycin were purchased from Selleck Chemicals, and dissolved in dimethyl sulfoxide and stored at −20 °C. Human breast epithelial cell lines (MDA-MB-453, BT-474, MCF-7, T47D, BT-549, BT-20, MDA-MB-435, MDA-MB-231, MDA-MB-468, and MCF-10A) were obtained from the American Type Culture Collection. BT-20, T47D, MDA-MB-231, MCF-7, MDA-MB-453, and MDA-MB-435 were routinely maintained in high-glucose DMEM. MDA-MB-468, BT-474, and BT-549 were routinely maintained in RPMI1640. MCF-10A was routinely maintained in MEBM plus 100 ng/ml cholera toxin. All media were supplemented with 10% fetal bovine serum and 1% penicillin–streptomycin except MEBM.

### Western blot and antibodies

Western blot were performed as previously described^22,31^. The antibodies used were as follows: Aurora-A (cell signaling, #91590), Phospho-Aurora-A (Thr288) (cell signaling, #3079), mTOR (cell signaling, #2983), Phospho-mTOR (Ser2448) (cell signaling, #5536), AKT (cell signaling, #4691), Phospho-AKT(Ser473) (cell signaling, #4060), ERK (cell signaling, #4695), Phospho-p44/42 MAPK (Erk1/2) (Thr202/Tyr204) (cell signaling, #4370), FLAG (Sigma-Aldrich, # F4042;), E-cadherin (cell signaling, #14472), p21 (cell signaling, #2947), ki-67 (cell signaling, #9449), PARP (cell signaling, #9532), cleavage Caspase-3 (cell signaling, #9661), and β-actin (cell signaling, #14793).

### Immunohistochemical staining

The breast cancer tissue arrays were used in our previous study^30^. Immunohistochemical staining of human TNBC TMAs or mice tumors was performed as described previously^50,51^. Briefly, the TMAs slides or mice tumor tissues were deparaffinized in xylene, rehydrated through graded alcohol, and then immersed in 3% hydrogen peroxide to block endogenous peroxidase activity. After an antigen retrieval process, the slides were incubated with the primary antibodies overnight at 4 °C in a moist chamber. Specimens were stained with DAB (3,3-diaminobenzidine; Dako, K5007) after being incubated with the secondary antibody (HRP-anti-Rabbit; Thermo Scientific, 31460). Finally, the sections were counterstained with hematoxylin, dehydrated and mounted.

The brown granules in cytoplasm of Aurora-A and p-mTOR were considered as positive staining. For the assessment of cytoplasmic staining, the staining intensity was scored as follows^32^: negative (score 0), bordering (score 1), weak (score 2), moderate (score 3), and strong (score 4). Staining extent was graded into five parts according to the percentage of elevated staining cells in the field: negative (score 0), 0–25% (score 1), 26–50% (score 2), 51–75% (score 3), and 76–100% (score 4). Aurora-A and p-mTOR expression were evaluated by combined assessing of staining intensity and extent. The merged overall score was subjected to further analysis.

### ATPlite cell proliferation assay

Cells were seeded into 96-well plates in triplicate. At different time after cell plating, cells were subjected to the ATPlite proliferation assay (Perkin-Elmer), according to the manufacturer’s instructions.

### Cell clonogenic assay

Cells were seeded into 60-mm dishes in triplicate, followed by incubation at 37 °C for 14–21 days and then the colonies were fixed with 10% acetic acid in methanol, stained with 0.05% methylene blue, and counted^51,52^.

### siRNAs and plasmid transfection

The siRNA sequences targeting Aur-A were used in our previous study; sequences targeting ERK1 (sc-29307) or 2 (sc-35335) were purchased from Santa Cruz and mixed together before use; sequence targeting mTOR is 5′-CUUCGAGACAUGA GUCAGCUUTT-3′; the sequence for the scrambled control siRNA is 5′-AUUGUAUGCGAUCGCAGACUU-3′^53,54^. The plasmids of vector control, FLAG-Aur-A-WT, and -D274A were constructed and used in our previous study^42^. Transfection of siRNAs or plasmids was carried out using Lipofectamine 2000 (Invitrogen) according to manufacturer’s instructions.

### Transwell migration assay

Transwell assay was performed as described previously^30^. After incubation, top cells were removed and bottom cells were fixed and stained with 4,6-diamidino-2-phenylindole (5 mg/mL) to visualize nuclei. The number of migrating cells in five fields was counted and the average number from each chamber was determined.

### In vivo xenograft model

All animal experiments were carried out according to a protocol approved by the University Committee for Use and Care of Animals. A total of 2 × 10^6^ MDA-MB-231 cells were mixed 1:1 with matrigel (BD biosciences, San Jose, CA) in a total volume of 0.2 ml and then injected subcutaneously into both flanks of nude mice, which were randomized into four groups (five mice for each group) and treated with vehicle, MLN8237 (30 mg/kg/days, every day, per gavage), rapamycin (3.75 mg/kg/days, 5 days a week, per gavage) or MLN8237 + rapamycin when the tumor size reached 100 mm^3^. Mice were followed up for tumor size, well-being, and body weight, and then killed when tumors in the control group reached an average of 1.5 cm in their largest dimension (21 days of treatment). Tumors were resected, weighed, and frozen or fixed in formalin and paraffin-embedded for immunohistochemical studies.

### Statistical analysis

Statistical analysis was performed using SPSS version 20.0 (SPSS Inc.). Student’s *t*-test was used to make a statistical comparison between groups. Both CalcuSyn software (Biosoft, Ferguson, MO, USA) and Jin’s formula were used to evaluate the synergistic effects of drug combinations as described previously^31,34^.

## Supplementary information

Supplemental Figure Legends

Supplemental Figures
