# Supplementary material for: Aurora-A/ERK1/2/mTOR axis promotes tumor progression in triple-negative breast cancer and dual-targeting Aurora-A/mTOR shows synthetic lethality
Source: Cell Death Dis. 2019 Aug 13;10(8):606. doi: 10.1038/s41419-019-1855-z (PMC6690898; doi:10.1038/s41419-019-1855-z)
Supplement: Supplementary file 1 — Supplemental Figure Legends [file 41419_2019_1855_MOESM1_ESM.doc]

**Supplemental Figure Legends**

**Figure S1 Aur-A promotes cell proliferation and invasion through activation of mTOR in TNBC cell lines**

(A) Inhibition of Aur-A decreases both p-Aur-A and p-mTOR in TNBC cells. Cells were incubated with indicated doses of MLN8237 (0, 0.25, 0.5, 1, and 2 μM) for 48h, followed by IB analysis. (B) Overexpression of Aur-A-WT increases p-mTOR, but -D274A dead mutant decreases p-mTOR. Cells were transfected with indicated plasmids and collected for IB assay after transfection. (C-E) Silencing Aur-A inhibits cell proliferation and suppresses migration by decreasing p-mTOR. Cells were transfected with siRNAs targeting Aur-A and then collected for IB assay (C), ATPlite assay (D), or transwell migration assay (E) (mean ± SD; ** P < 0.01). (F-H) Overexpression of Aur-A promotes cell proliferation and migration by activating mTOR and silencing mTOR abrogates. Cells were transfected with vector control or FLAG-tagged Aur-A firstly and then silenced by using siRNAs targeting control or mTOR, followed by IB (F), ATPlite (G), and transwell migration (H) assays (mean ± SD; * P < 0.05; ** P < 0.01)

**Figure S2 Inhibition of ERK1/2 decreased p-mTOR but inhibition of mTOR failed to change the level of p-ERK1/2.**

Cells were treated with DMSO, Rapamycin (0.1 µM), or U0126 (5 µM) for 48h, followed by IB analysis.

**Figure S3 Dual inhibition of Aur-A and Rapamycin induced apoptosis in TNBC cells.**

Cells were treated with MLN8237 (0.1 μM) or Rapamycin (0.1 μM), or combination of the two compounds, followed by IB assay with indicated antibodies.
