# Supplementary figures and images for: Aurora-A/ERK1/2/mTOR axis promotes tumor progression in triple-negative breast cancer and dual-targeting Aurora-A/mTOR shows synthetic lethality
Source: Cell Death Dis. 2019 Aug 13;10(8):606. doi: 10.1038/s41419-019-1855-z (PMC6690898; doi:10.1038/s41419-019-1855-z)

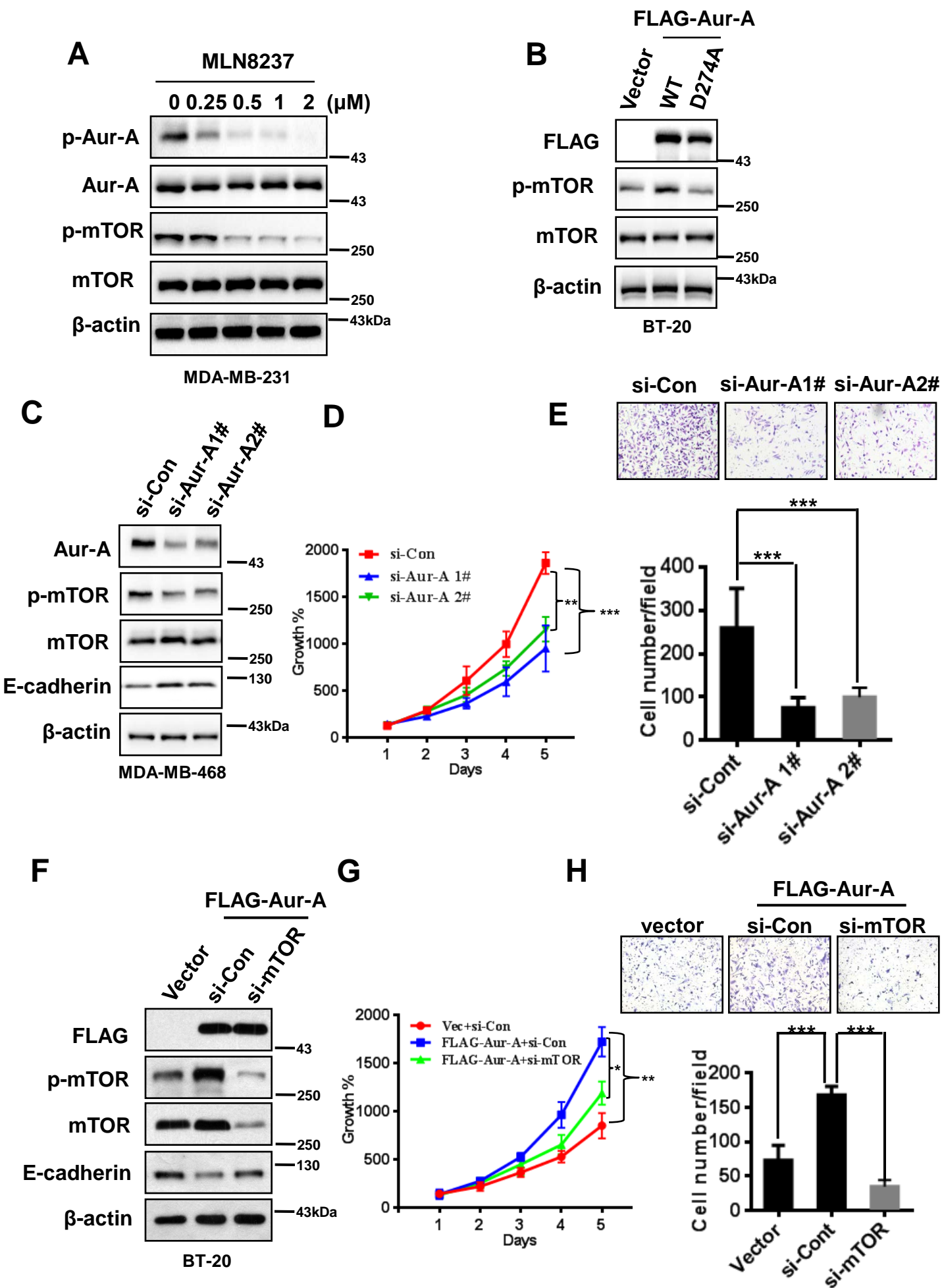

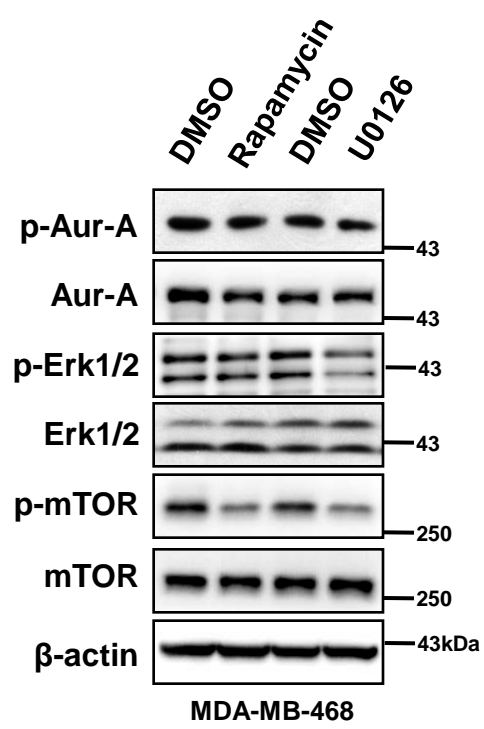

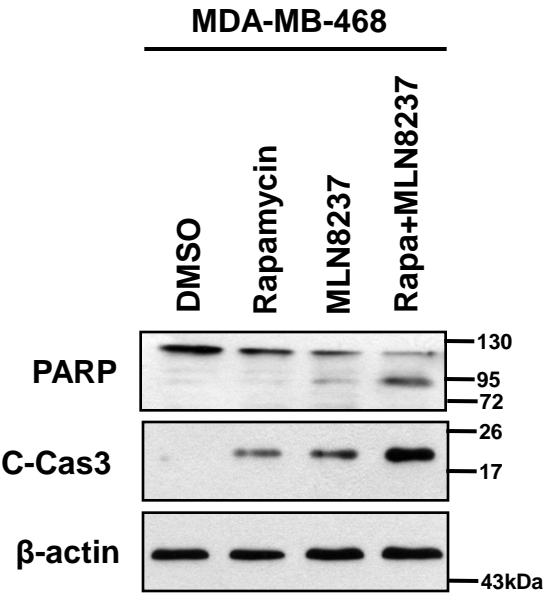

Supplement: Supplementary file 2 — Supplemental Figures [file 41419_2019_1855_MOESM2_ESM.pdf]
